# Comparative effectiveness of daytime versus nighttime paravertebral block analgesia on intraoperative hemodynamics in older lung cancer patients: a target trial emulation study

**DOI:** 10.3389/fmed.2026.1752499

**Published:** 2026-08-19

**Authors:** Qiang Wang, Lin Zhuo, Yue Zhang, Bowen Zhang, Kaibing Yang, Shijing Wei, Jingchao Yang, Xinwei Ma, Xiaolin Han, Hui Zheng, Yang Xu

**Affiliations:** 1 Department of Anesthesiology, National Cancer Center/National Clinical Research Center for Cancer/Cancer Hospital, Chinese Academy of Medical Sciences and Peking Union Medical College, Beijing, China; 2 Research Center of Clinical Epidemiology, Peking University Third Hospital, Beijing, China; 3 Key Laboratory of Epidemiology of Major Diseases (Peking University), Ministry of Education, Beijing, China; 4 Department of Pharmacy Administration and Clinical Pharmacy, School of Pharmaceutical Sciences, Peking University Health Science Center, Beijing, China; 5 Information Management Center, National Cancer Center/National Clinical Research Center for Cancer/Cancer Hospital, Chinese Academy of Medical Sciences and Peking Union Medical College, Beijing, China; 6 Department of Medical Epidemiology and Biostatistics, Karolinska Institutet, Stockholm, Sweden

**Keywords:** intraoperative hemodynamics, lung cancer surgery, older patients, paravertebral block, target trial emulation

## Abstract

Surgery remains the mainstay treatment for older patients with lung cancer, and paravertebral block (PVB) is recommended for perioperative analgesia. With the increasing surgical demand, a growing proportion of elective thoracic procedures in China are scheduled at night. Circadian variation, prolonged fasting, and resource limitations may affect intraoperative hemodynamics. However, the comparative hemodynamic safety of daytime versus nighttime PVB has not been systematically investigated. We emulated a target trial using routinely collected anesthesia and electronic medical records from the Cancer Hospital Chinese Academy of Medical Sciences (Oct-2020 to Jun-2022). Patients aged ≥65 years undergoing lung cancer surgery with PVB were classified into daytime (08:00–16:59) or nighttime (17:00–7:59) groups. Confounding was addressed with stabilized inverse probability of treatment weighting. The primary outcomes were intraoperative hypotension (systolic blood pressure <90 mmHg or diastolic blood pressure <60 mmHg) and bradycardia (heart rate <60 bpm). Secondary outcomes included intravenous fluid volume and vasoactive drug use. Time-to-first and recurrent events were assessed using weighted Cox and Poisson regression. Sensitivity analyses included propensity score matching, restricting to patients with PVB after general anesthesia, and adding surgical type as a covariate. Among 637 patients (479 daytime, 158 nighttime), nighttime PVB was associated with more recurrent hypotension (adjusted-incidence rate ratio [IRR]: 1.04; 95% confidence interval [CI], 1.01–1.09), but no difference in bradycardia (hazard ratio [HR]: 1.11, 95% CI, 0.90–1.36; IRR 1.02, 95%CI 0.98–1.05). Intravenous fluid use was lower at night (mean difference −53.20 mL; 95% CI, −104.36 to −2.04). Findings were robust across sensitivity analyses, with stronger associations in women and patients with hypertension or arrhythmia. We conclude that in older patients undergoing lung cancer surgery, nighttime PVB was linked to a modestly higher burden of hypotension and lower fluid administration compared with daytime PVB. Vigilant monitoring and optimized perioperative management are warranted, and multicenter studies should validate these findings.

## Background

Lung cancer remains the most frequently diagnosed malignancy and the leading cause of cancer-related mortality globally (1), accounting for over 2.5 million new cases and approximately 1.8 million deaths globally in 2022. The incidence is particularly high among older patients, with over two-thirds of cases occurring in individuals aged 65 years or above (2, 3). Surgical resection continues to represent the cornerstone of curative treatment, yet perioperative outcomes in this population remain closely tied to the quality of analgesia and hemodynamic stability achieved during anesthesia. Paravertebral block (PVB) has become a widely adopted technique in thoracic anesthesia and is strongly recommended by the European Society of Regional Anaesthesia and Pain Therapy and other professional societies (4, 5). Compared with systemic opioid-based analgesia, PVB provides superior pain relief, reduces opioid consumption, lowers pulmonary complication rates, and facilitates early mobilization and shorter hospital stays (6–8). Consequently, PVB has become a standard adjunct for perioperative management in lung cancer surgery.

In recent years, many tertiary centers in China and elsewhere have faced rapidly growing surgical demands due to population aging and the rising incidence of cancer. As a result, an increasing number of elective thoracic procedures are scheduled not only during the day but also at night (9–12). While this practice helps reduce preoperative delays, it also introduces new physiological and organizational challenges. Circadian rhythms exert profound effects on cardiovascular function, including fluctuations in blood pressure, heart rate, vascular tone, and hormonal regulation (13, 14). Additionally, the potentially longer preoperative fasting time associated with nighttime elective surgeries, as well as the increased preoperative anxiety due to long daytime waiting for nighttime surgery, may further impact the function of the circulatory system (15). Older patients may be especially vulnerable due to reduced baroreflex sensitivity, diminished cardiac reserve, increased arterial stiffness, and impaired autonomic regulation (16, 17). Importantly, PVB itself can attenuate sympathetic outflow by blocking the paravertebral sympathetic chain, which in the context of older patients undergoing nighttime surgery may compound the risk of hypotension or bradycardia (18). Taken together all above factors suggest that the hemodynamic effects of PVB may differ by time of day.

Despite these concerns, no prior study has directly compared the hemodynamic consequences of daytime versus nighttime PVB in older patients undergoing lung cancer surgery. To address this evidence gap, we emulated a target trial using hospital-based electronic records to evaluate whether PVB timing influences intraoperative hypotension, bradycardia, and related management strategies, to provide robust real-world evidence to inform clinical decision-making and optimize perioperative care for this rapidly expanding patient population.

## Methods

### Study design and data source

We emulated a target trial using routinely collected electronic medical records (EMRs) and anesthesia records from the Cancer Hospital Chinese Academy of Medical Sciences (CHCAMS) (Beijing, China). The study period was October 19, 2020, to June 28, 2022. This research was approved by the institutional ethics committee (approval number 24/108–4,388), with waiver of informed consent due to the use of de-identified data. The study was prospectively registered at the Chinese Clinical Trial Registry (ChiCTR2400081812).

### Target trial specification and emulation

Following the target trial emulation framework (19), we first specified the protocol of a target trial that would evaluate the effects of daytime versus nighttime PVB analgesia on the hemodynamics of older patients undergoing lung cancer surgery. The key components of the target trial protocol and emulation process were presented in detail below and in Supplementary Table S1.

### Eligibility criteria

Our cohort included all older patients (≥65 years) undergoing the lung cancer surgery who received PVB analgesia. The time of undergoing PVB analgesia was the index time (T_0_). Patients with missing information on height, weight, or hemodynamics before T_0_ were excluded. Detailed definitions of all inclusion/exclusion criteria were presented in Supplementary Table S2.

After applying eligibility criteria, a total of 637 elderly individuals were finally included (Supplementary Figure S1).

### Treatment strategies

We compared two treatment strategies: “*start a daytime PVB analgesia (8:00–16:59) and continue use during follow-up*” *vs.* “*start a nighttime PVB analgesia (17:00–7:59) and continue use during follow-up*.” We defined 17:00 as the cutoff between daytime and nighttime because it marks the onset of staff shift changes in the operating room, a period associated with changes in personnel continuity and clinical resource allocation.

### Treatment assignment

Patients who received daytime or nighttime PVB analgesia during the study period were assigned to the treatment arm.

### Outcomes and study covariates

The primary outcomes were hypotension, defined as the presence of systolic blood pressure (SBP) < 90 mmHg or diastolic blood pressure (DBP) < 60 mmHg, and bradycardia, defined as heart rate <60 bpm. We computed both the time to the first-recorded event (i.e., first occurrence), and the incidence rate over time (i.e., recurrence). In addition, we also computed the proportion of time for hypotension and bradycardia (Supplementary Figure S2).

The secondary outcomes included intravenous fluid use (crystalloids, colloids, and total volume), as well as the use of vasopressors and positive chronotropic agents. Definitions of each outcome were detailed in Supplementary Table S3.

The following covariates were extracted and considered in our analysis for confounding adjustment: demographics (age, sex, height, and weight), hemodynamics before T_0_ (SBP, DBP, and heart rate), American Society of Anesthesiologists (ASA) physical status (I, II, and III), history of comorbidities (hypertension, arrhythmia, hyperthyroidism, and hypothyroidism), the order of general anesthesia (GA) and PVB analgesia (GA after PVB, PVB after GA), medication use for anesthesia (sedative-hypnotics, analgesics, and skeletal muscle relaxants). These variables were selected based on prior literature.

### Start and end of follow-up

Patients were monitored from T_0_ until the earlier occurrence of the first-recorded primary outcome, or until exiting the operating room. For other outcomes, data collection continued until patients exited the operating room.

### Causal contrast

Our main analysis used an intention-to-treat (ITT) approach, which analyses patients according to their original treatment assignment at baseline, regardless of treatment discontinuation or switching during follow-up.

### Statistical analyses

Baseline characteristics were summarized, and presented as mean ± standard deviation (SD) or medians with interquartile ranges (IQR) for continuous variables, depending on the distribution, and as numbers and percentages for categorical variables.

We used inverse probability of treatment weighting (IPTW) to control for baseline confounding (20, 21). Using a multivariable logistic regression model, we estimated the probability of daytime versus nighttime PVB (propensity score, PS) as a function of the baseline covariates listed above. Patients in the nighttime PVB group were weighted by 1/PS and in the daytime PVB group by 1/(1-PS). Weights were stabilized by adding the marginal probability of daytime or nighttime PVB to the numerator of the weights (Supplementary Figure S3). To assess the covariate balance between the two groups, we computed standardized absolute mean differences (SAMDs) both before and after the weighting, using a SAMD of >0.1 as the threshold for meaningful imbalance.

We used Poisson regression to estimate crude incidence rates per person-hour for the first hypotension and bradycardia event and all hypotension and bradycardia events during the follow-up. Weighted Cox proportional hazards regression was used to estimate hazard ratios (HRs) for the first hypotension and bradycardia event. Incidence rate ratios (IRRs) of recurrent hypotension and bradycardia events were estimated by including the above weights in a modified Poisson regression model. Weighted cumulative incidence curves and the weighted Dong-Yasui estimator were used to compare the incidences of first hypotension and bradycardia event and mean cumulative hypotension and bradycardia counts (22) between the two groups. Weighted linear regression was used to estimate the mean difference for the proportion of time for hypotension, proportion of time for bradycardia, and intravenous fluid use. The odds ratio (OR) of medication use (vasopressors and positive chronotropic agents) was estimated by weighted logistic regression.

For all weighted analyses, confidence intervals (CI) were obtained by robust variance estimation to account for uncertainty around weights.

### Subgroup and sensitivity analyses

Subgroup analyses were performed to test for potential effect modification of age (≥70 years, <70 years), sex, presence or absence of baseline hypertension, and presence or absence of baseline arrhythmia.

To test the robustness of our study findings, the following sensitivity analyses were performed: (1) To evaluate the strength of the association between unmeasured confounders and the group assignment, as well as the outcomes, to explain the observed treatment effect on our study results, we calculated an E-value; (2) To control the influence of the order of PVB analgesia and GA on study outcomes, we restricted our study population to patients who received PVB after GA; (3) Instead of estimating the average treatment effect (ATE) in the total population, we estimated the average treatment effect in the treated (ATT) population. 1:3 propensity score matching by the “nearest-neighbor” approach with a caliper of 0.05 was used and PSs were calculated as in the main analysis; (4) To further mitigate confounding, we added different surgical types (thoracotomy or video thoracoscopy) as a covariate to the original model.

All analyses were conducted with R 4.3.1 (R Foundation for Statistical Computing, Vienna, Austria).

## Results

### Patient characteristics

**Figure 1:**
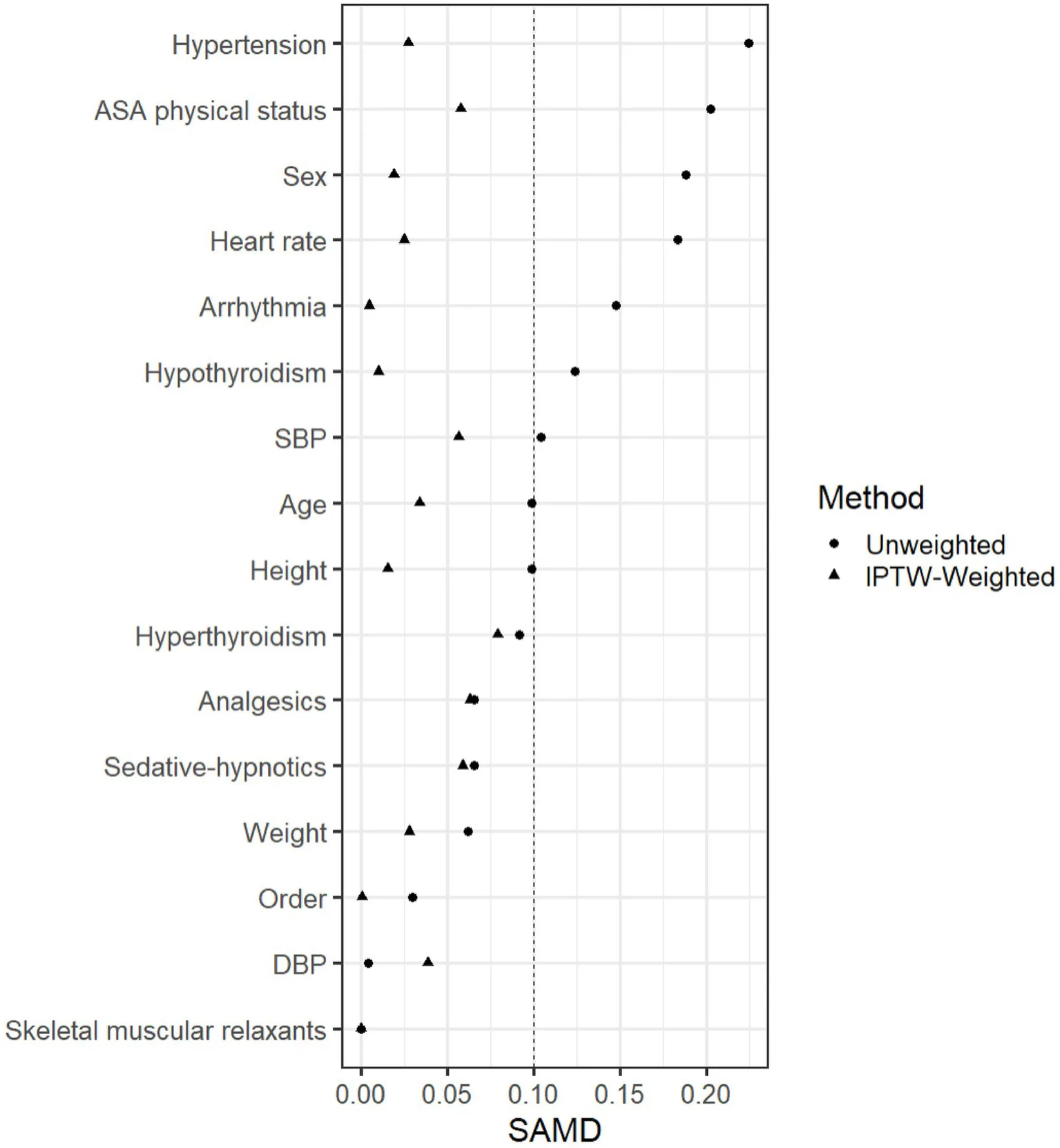
Balance of baseline characteristics before and after inverse probability of treatment weighting.

The vast majority of patients underwent lobectomy (635/637, 99.7%), with only two patients (2/637, 0.3%) undergoing pneumonectomy (both in the daytime PVB group). 637 patients were included in the analyses, with 479 (75.2%) patients receiving daytime PVB analgesia and 158 (24.8%) patients receiving nighttime PVB analgesia (Supplementary Figure S1). The nighttime PVB analgesia group had more female patients (63.3% versus 54.1%), similar SBP (145.5 mmHg versus 143.0 mmHg), and heart rate (75 bpm versus 72 bpm) compared to the daytime PVB analgesia group. Moreover, the nighttime PVB analgesia group had a lower prevalence of hypertension (31.0% versus 41.8%), arrhythmia (12.7% versus 18.0%), and a slightly higher prevalence of hypothyroidism (1.3% versus 0.2%) than the daytime PVB analgesia group. There were also differences in the composition ratios of ASA physical status between the two groups (daytime PVB analgesia versus nighttime PVB analgesia; [I, 0% versus 0.6%; II, 92.3% versus 95.6%, and III, 7.7% versus 3.8%]). Baseline characteristics before and after weighting were reported in Supplementary Table S4 and were all well-balanced after weighting (Figure 1).

### Primary outcomes: hypotension and bradycardia

**Figure 2:**
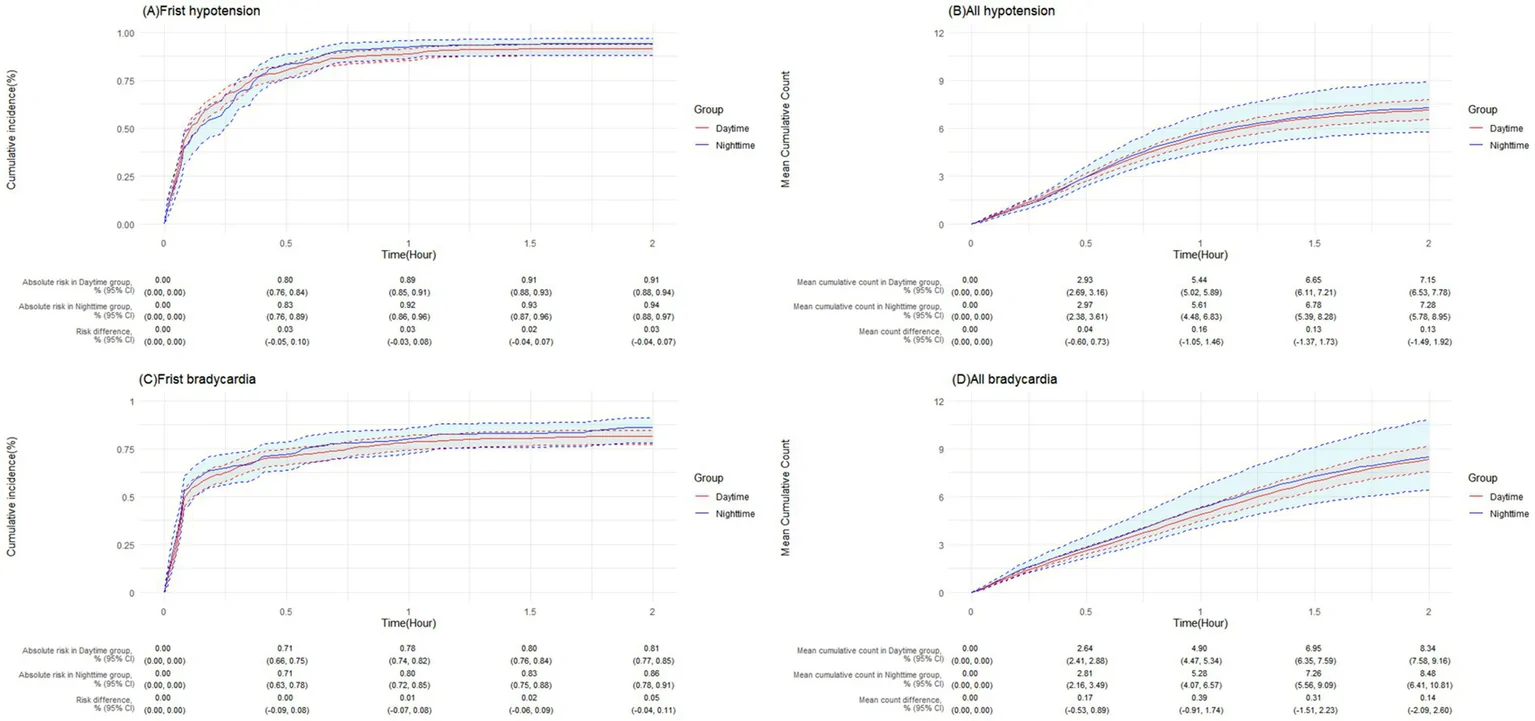
Weighted cumulative incidence curves for **(A)** First hypotension outcome, **(B)** All hypotension outcome, **(C)** First bradycardia outcome, and **(D)** All bradycardia outcome for daytime PVB versus nighttime PVB. Dotted lines represent 95% confidence intervals (CIs).

Over a median follow-up of 1.90 (IQR, 1.45, 2.45) hours, 569 patients (422 daytime PVB analgesia and 147 nighttime PVB) experienced at least one hypotension event (adjusted-HR: 0.99; 95% CI, 0.82, 1.19). We observed a total of 3,678 hypotension episodes for 479 daytime PVB analgesia patients and 1,170 hypotension episodes for 158 nighttime PVB analgesia patients, accounting for incidence rates of 3.84 (95% CI, 1.53, 8.52) and 3.90 (95% CI, 1.57, 8.61) episodes per person-hour, respectively. The adjusted IRR of nighttime PVB analgesia versus daytime PVB analgesia was 1.04 (95% CI, 1.01, 1.09) (Table 1). Weighted cumulative incidence curves and weighted mean cumulative count curves of nighttime versus daytime PVB analgesia both showed an early separation, and the separation was sustained over the whole follow-up (Figure 2 Panel A and B). From the curves, a 2-h absolute risk difference of 0.03 (−0.04, 0.07) was estimated for the first hypotension, while a 2-h mean count difference of 0.13 (−1.49, 1.92) was estimated for all hypotension.

**Table 1:** Number, rate of and proportion of time for hypotension/bradycardia events in daytime PVB versus nighttime PVB.

Outcomes	Exposure	No. of persons	No. of events	Median follow-up [IQR], hr	Person-hour	Incidence rate per p-hr (95% CI)	Crude HR (95% CI)	Adjusted HR (95% CI)
Panel A. hypotension/bradycardia occurrence (time to first event)								
First hypotension	Daytime PVB	479	422	1.93 (1.47, 2.48)	958	0.44 (0.10, 2.34)	Ref.	Ref.
	Nighttime PVB	158	147	1.78 (1.37, 2.35)	300	0.49 (0.11, 2.48)	0.99 (0.82,1.20)	0.99 (0.82, 1.19)
First bradycardia	Daytime PVB	479	382	1.93 (1.47, 2.48)	958	0.40 (0.09, 2.22)	Ref.	Ref.
	Nighttime PVB	158	130	1.78 (1.37, 2.35)	300	0.43 (0.09, 2.32)	1.00 (0.82, 1.21)	1.11 (0.90, 1.36)

Outcomes	Exposure	No. of persons	No. of events	Median follow-up [IQR], hr	Person-hour	Incidence rate per p-hr (95% CI)	Crude IRR (95% CI)	Adjusted IRR (95% CI)
Panel B. hypotension/bradycardia recurrence (rate of multiple events per patient)								
All hypotension	Daytime PVB	479	3,678	1.93 (1.47, 2.48)	958	3.84 (1.53, 8.52)	Ref.	Ref.
	Nighttime PVB	158	1,170	1.78 (1.37, 2.35)	300	3.90 (1.57, 8.61)	1.04 (1.01, 1.08)	1.04 (1.01, 1.09)
All bradycardia	Daytime PVB	479	4,558	1.93 (1.47, 2.48)	958	4.76 (2.06, 9.89)	Ref.	Ref.
	Nighttime PVB	158	1,410	1.78 (1.37, 2.35)	300	4.70 (2.02, 9.81)	0.98 (0.95, 1.02)	1.02 (0.98, 1.05)

Outcomes	Exposure	No. of persons	Mean (SD)	Crude mean difference (95% CI)	Adjusted mean difference (95% CI)
Panel C. proportion of time for hypotension/bradycardia					
Proportion of time for hypotension	Daytime PVB	479	0.35 (0.28)	Ref.	Ref.
	Nighttime PVB	158	0.36 (0.28)	0.01 (−0.04, 0.06)	0.01 (−0.04, 0.06)
Proportion of time for bradycardia	Daytime PVB	479	0.41 (0.35)	Ref.	Ref.
	Nighttime PVB	158	0.40 (0.34)	−0.003 (−0.064, 0.059)	0.01 (−0.05, 0.07)

PVB, paravertebral block; CI, confidence interval; p-hr, person-hour; HR, hazard ratio; IRR, incidence rate ratio; SD, standard deviation.

Number of events, person-hour, incidence rates and mean were calculated in the unweighted population.

For bradycardia, no differences were observed between groups. The HR for first bradycardia was 1.11 (95% CI 0.90–1.36), and the IRR for recurrent events was 1.02 (95% CI 0.98–1.05) (Table 1). The trends of the two curves were consistent with the trends of hypotension outcomes, both showing an early separation that persisted throughout the entire follow-up period, with a 2-h absolute risk difference of 0.05 (−0.04, 0.11) and mean count difference of 0.14 (−2.09, 2.60) (Figure 2 Panel C and D).

The mean proportion of intraoperative time spent in hypotension or bradycardia did not differ significantly between groups (hypotension: adjusted mean difference 0.01; 95% CI –0.04 to 0.06; bradycardia: 0.01; 95% CI –0.05 to 0.07).

### Secondary outcomes: intravenous fluid and medication use

The unadjusted mean total intravenous fluid volume was 1,158 mL in the daytime PVB group and 1,096 mL in the nighttime PVB group. Patients receiving nighttime PVB were given less intravenous fluid overall than those in the daytime group (adjusted mean difference: −53.20 mL; 95% CI, −104.36, −2.04; Table 2). Differences in crystalloid and colloid administration were not statistically significant [(adjusted mean difference: −24.30; 95% CI, −72.12, 23.52) and colloidal use (adjusted mean difference: −21.60; 95% CI, −56.88, 13.68)].

**Table 2:** Mean of intravenous fluid and medication use in daytime PVB versus nighttime PVB.

Outcomes	Exposure	No. of persons	Mean (SD)	Crude mean difference (95% CI)	Adjusted mean difference (95% CI)
Panel A. intravenous fluid use					
Crystalline, mL	Daytime PVB	479	1059.71 (268)	Ref.	Ref.
	Nighttime PVB	158	1026.58 (260)	−33.13 (−80.43, 14.18)	−24.30 (−72.12, 23.52)
Colloidal, mL	Daytime PVB	479	91.86 (202)	Ref.	Ref.
	Nighttime PVB	158	69.62 (174)	−22.24 (−54.92, 10.44)	−21.60 (−56.88, 13.68)
Total volume, mL	Daytime PVB	479	1158.46 (293)	Ref.	Ref.
	Nighttime PVB	158	1096.20 (231)	−62.25 (−106.94, −17.56)	−53.20 (−104.36, −2.04)

Outcomes	Exposure	No. of persons	No. of events (%)	Crude OR (95% CI)	Adjusted OR (95% CI)
Panel B. medication use					
Vasopressors	Daytime PVB	479	259 (54.07)	Ref.	Ref.
	Nighttime PVB	158	71 (44.94)	0.69 (0.48, 0.99)	0.75 (0.52, 1.08)
Positive chronotropic agents	Daytime PVB	479	3 (0.01)	/	/
	Nighttime PVB	158	0 (0)	/	/

PVB, paravertebral block; CI, confidence interval; OR, odds ratio; SD, standard deviation.

Number of events, and mean were calculated in the unweighted population.

IPTW, inverse probability of treatment weighting; SAMD, standardized absolute mean difference; ASA, American Society of Anesthesiologists; SBP, systolic blood pressure; DBP, diastolic blood pressure; Order: the order of paravertebral block analgesia and general anesthesia.

In our analysis, we did not find a difference between the time of undergoing PVB analgesia and vasopressors use (adjusted-OR: 0.75; 95% CI, 0.52, 1.08) (Table 2). The effect value of the use of positive chronotropic agents was not reported because of the limited number of users (*n* = 3).

### Subgroup and sensitivity analyses

Subgroup analyses suggested that the association between nighttime PVB and recurrent hypotension was more pronounced among patients aged <70 years (1.11 [95% CI, 1.05, 1.17]), female (1.10 [95% CI, 1.05, 1.16]), and those with baseline with hypertension (1.24 [95% CI, 1.17, 1.32]), or arrhythmia (1.23 [95% CI, 1.11, 1.36]). For bradycardia, modest increases were observed in patients aged ≥70 years (1.08 [95% CI, 1.03, 1.14]), female (1.11 [95% CI, 1.06, 1.16]) or patients baseline with hypertension (1.12 [95% CI, 1.06, 1.18]), though confidence intervals were wide and overlapped the null (Supplementary Table S5).

The robustness of findings was confirmed in multiple analyses. The point estimate of the E-value for all hypotension outcomes was 1.26, with a lower confidence limit of 1.06, indicating that a moderately strong unmeasured confounder would be needed to fully explain the observed effect. When limiting our study population to PVB after GA analysis, the primary outcomes in both treatment groups during follow-up were consistent with the main analyses (Supplementary Table S6). For PS matching, we included 357 patients undergoing daytime PVB and 148 patients undergoing nighttime PVB. The baseline characteristics of these patients were summarized in Supplementary Table S7 and all were well-balanced after matching (Supplementary Figure S4). ATT estimates were similar to ATE estimates in main analyses by IPTW (Supplementary Table S8). We found that patients with video thoracoscopy had a higher risk of all hypotension undergoing nighttime PVB (adjusted-IRR: 1.06; 95% CI, 1.02, 1.11) (Supplementary Table S9).

## Discussion

### Principal findings

This study emulated a target trial using hospital-based electronic medical records from the leading cancer hospital in China—the largest cancer treatment center in Asia—to evaluate the effect of different timings of PVB analgesia on intraoperative hemodynamic stability in older patients undergoing lung cancer surgery. Our results show that nighttime PVB was associated with a modest but statistically significant increase in all hypotension, consistenting across subgroups, including age <70 years, females, and those with baseline hypertension. Although nighttime PVB was associated with a statistically significant increase in recurrent hypotension episodes, the observed effect size was modest (adjusted IRR 1.04). Nevertheless, because intraoperative hypotension is a common and potentially preventable adverse event in older patients, even a modest increase in recurrent hypotension may warrant greater vigilance in perioperative hemodynamic monitoring, particularly among patients with additional risk factors. By contrast, no significant differences were observed in time-to-first hypotension or bradycardia outcomes. While the main analysis showed no difference in all bradycardia events, subgroup analysis revealed an increased nighttime PVB-related bradycardia risk in patients aged 70 years and above, females, and those with hypertension. Meanwhile, the results showed a higher risk of all hypotension with nighttime PVB during thoracoscopic surgery.

In this study, we defined nighttime as starting at 17:00 based on multiple practical and clinical considerations. 17:00 was chosen because it coincides with the time when anesthesia, nursing, and surgical teams rotate for dinner breaks. This temporary adjustment in staffing may cause brief disruptions in workflow and team coordination, subtly impacting procedural efficiency and intraoperative management (23). Additionally, hospital resources and medical support tend to be more limited after 17:00, with reduced staffing levels, decreased immediate availability of specialized personnel, and fewer on-site consultants (24), all of which may potentially affect the maintenance and management of intraoperative hemodynamic stability. As the day progresses, accumulated physical fatigue among healthcare providers can further contribute to decreased attention and slower reaction times (25). Considering these factors, setting 17:00 as the threshold allows for a meaningful comparison of PVB analgesic outcomes under varying clinical conditions, providing insights into the potential impact of different times of day and night on intraoperative hemodynamics.

Several plausible mechanisms may underlie the higher incidence of hypotension observed during nighttime PVB compared with daytime PVB. Firstly, due to the unpredictable start time of nighttime surgeries, patients may experience prolonged fasting periods, which can lead to dehydration and reduced circulatory volume, thereby increasing the risk of hypotension following PVB (26, 27). This risk is even more pronounced in older patients, as age-related physiological changes can impair circulatory system function and disrupt fluid balance (28). Secondly, the body’s natural circadian rhythm, which includes lower blood pressure at night, may contribute to hypotension after PVB in older patients, including both normotensive and hypertensive individuals undergoing nighttime PVB analgesia (29). This is particularly relevant considering the critical roles of key hormones, such as melatonin and cortisol, in the regulation of blood pressure (30). During the night, melatonin levels rise, promoting vasodilation and potentially contributing to the observed lower blood pressure in patients undergoing nighttime PVB (31, 32). In contrast, cortisol, which typically peaks in the morning, is at its lowest during the night (33). The reduced cortisol levels during nighttime surgery may impair the body’s ability to maintain vascular tone, further increasing the risk of hypotension (34). These hormonal fluctuations, combined with the effects of PVB, may help explain the higher incidence of hypotension observed in older lung cancer patients after nighttime PVB. Thirdly, in older patients with lung cancer undergoing elective nighttime surgery, a prolonged preoperative waiting period may contribute to increased anxiety and elevated sympathetic nervous system activity (35). We frequently observe this phenomenon in our daily clinical practice. However, after the administration of PVB analgesia, the blockade of the sympathetic nerve chain can cause a sudden reduction in sympathetic tone, potentially leading to a more pronounced decrease in blood pressure, especially in patients with pre-existing sympathetic activation (36). Fourthly, our study found that total intravenous fluid administration was lower in older lung cancer patients who received nighttime PVB. This may be attributable to human factors, such as physical or mental fatigue of the anesthesia team and decreased vigilance in fluid management during nighttime procedures, potentially resulting in suboptimal perioperative fluid administration.

The increased risk of hypotension and bradycardia observed in older female patients undergoing nighttime PVB is likely attributable to postmenopausal estrogen deficiency (37), which impairs the autonomic regulation of blood pressure and heart rate, rendering them more vulnerable to these hemodynamic disturbances (38, 39). Additionally, older patients with baseline hypertension are at increased risk of hypotension and bradycardia after nighttime PVB (40). This may be attributed to the fact that older lung cancer patients with concomitant hypertension often exhibit cardiovascular system impairments (38), including endothelial dysfunction (39), myocardial hypertrophy, and decreased myocardial contractile function (40). Moreover, older lung cancer patients with hypertension often take antihypertensive medications, including beta-blockers, calcium channel blockers and angiotensin-converting enzyme inhibitors (41). These drugs usually have negative chronotropic effects, and also decrease cardiac output and peripheral vascular resistance (42). Furthermore, patients with baseline arrhythmia are more prone to hypotension following nighttime PVB. This is most likely due to the fact that arrhythmia can affect the heart’s ability to effectively pump blood, potentially leading to decreased cardiac output and subsequent hypotension after PVB (43, 44).

### Strengths and limitations of this study

This study employed an emulated target trial framework combined with a stabilized IPTW approach to control for measured confounders, demonstrating a methodological rigor comparable to randomized controlled trials while avoiding ethical and safety concerns. We specifically focused on older patients with lung cancer, who are often excluded from randomized trials because of their complex medical conditions, including a higher prevalence of chronic comorbidities and organ dysfunction. Propensity score matching (PSM) was also employed to validate our findings, and the sequence of GA and PVB analgesia was adjusted to assess the robustness of the results.

Despite these strengths, residual and unmeasured confounding remains an inherent limitation of this observational study. Although we adopted a target trial emulation framework combined with IPTW to minimize confounding by measured variables, and the E-value indicated that an unmeasured confounder would need to increase the likelihood of both nighttime PVB analgesia and recurrent hypotension by 1.26-fold to fully explain the observed association, unmeasured confounding cannot be completely excluded. In particular, several clinically relevant perioperative factors, including the duration of preoperative fasting, details of intraoperative anesthetic management, anesthesiologists’ experience, workload, staffing levels, surgeons’ decisions regarding surgical timing, patient frailty, and diabetes-related autonomic neuropathy, were not available in our database and therefore could not be incorporated into the propensity score model. Furthermore, our study focused exclusively on intraoperative hemodynamic outcomes. Although recurrent intraoperative hypotension has been associated with adverse postoperative events in previous studies, we did not evaluate whether the observed increase translated into clinically meaningful outcomes such as myocardial injury, acute kidney injury, pulmonary complications, intensive care unit admission, prolonged hospital stay, or mortality. Additionally, this study was conducted at a single high-volume tertiary cancer center and included only older patients undergoing lung cancer surgery with PVB. Consequently, our findings may not be directly generalizable to younger patients, other surgical populations, different regional anesthesia techniques, or healthcare systems with different perioperative practices and operating room organization. Future multicenter studies incorporating more detailed perioperative management variables together with clinically relevant postoperative outcomes are required to confirm these findings and determine their clinical importance.

## Conclusion

This cohort study of older patients undergoing PVB analgesia for lung cancer surgery indicates that nighttime PVB analgesia is associated with a higher risk of all hypotension and lower intravenous fluid use compared to daytime PVB analgesia. These findings highlight the importance of vigilant monitoring, optimized fluid management and promptly appropriate use of vasopressor medications during nighttime PVB analgesia to mitigate the risk of hypotension. Future multicenter studies, ideally integrating granular anesthesia practice data and postoperative outcomes, are needed to confirm these findings and to inform evidence-based guidelines for perioperative care across different times of day.

## Data Availability

The data analyzed in this study is subject to the following licenses/restrictions: the data, analytic methods, and study materials that support the findings of this study are available upon request from the corresponding authors. The data are not publicly available, due to privacy or ethical restrictions. Requests to access these datasets should be directed to QW, wqzjbd001@163.com.

## Glossary

PVB: Paravertebral block
EMRs: Electronic medical records
CHCAMS: Cancer Hospital Chinese Academy of Medical Sciences
SBP: Systolic blood pressure
DBP: Diastolic blood pressure
IPTW: Inverse probability of treatment weighting
PSM: Propensity score matching
PS: Propensity score
ASA: American Society of Anesthesiologists
GA: General anesthesia
SAMD: Standardized absolute mean difference
SD: Standard deviation
IQR: Interquartile range
CI: Confidence interval
p-hr: Person-hour
HR: Hazard ratio
OR: Odds ratio
IRR: Incidence rate ratio
ATT: Average treatment effect in the treated
ATE: Average treatment effect
