# Correction: Comparative effectiveness of daytime versus nighttime paravertebral block analgesia on intraoperative hemodynamics in older lung cancer patients: a target trial emulation study

**DOI:** 10.3389/fmed.2026.2004550

**Published:** 2026-10-13

**Authors:** Qiang Wang, Lin Zhuo, Yue Zhang, Bowen Zhang, Kaibing Yang, Shijing Wei, Jingchao Yang, Xinwei Ma, Xiaolin Han, Hui Zheng, Yang Xu

**Affiliations:** 1 Department of Anesthesiology, National Cancer Center/National Clinical Research Center for Cancer/Cancer Hospital, Chinese Academy of Medical Sciences and Peking Union Medical College, Beijing, China; 2 Research Center of Clinical Epidemiology, Peking University Third Hospital, Beijing, China; 3 Key Laboratory of Epidemiology of Major Diseases (Peking University), Ministry of Education, Beijing, China; 4 Department of Pharmacy Administration and Clinical Pharmacy, School of Pharmaceutical Sciences, Peking University Health Science Center, Beijing, China; 5 Information Management Center, National Cancer Center/National Clinical Research Center for Cancer/Cancer Hospital, Chinese Academy of Medical Sciences and Peking Union Medical College, Beijing, China; 6 Department of Medical Epidemiology and Biostatistics, Karolinska Institutet, Stockholm, Sweden

**Keywords:** intraoperative hemodynamics, lung cancer surgery, older patients, paravertebral block, target trial emulation

The funder ‘National High Level Hospital Clinical Research Funding and Beijing Hope Run Special Fund of Cancer Foundation of China’, [No. LC2022A05] to Qiang Wang was erroneously omitted.

The funder ‘Beijing Hope Run Special Fund of Cancer Foundation of China’, [No. LC2022A05] to Qiang Wang was erroneously included.

The correct funding statement reads:

’Supported by National High Level Hospital Clinical Research Funding and Beijing Hope Run Special Fund of Cancer Foundation of China (No. LC2022A05)’

The original version of this article has been updated.

