# Supplementary material for: Comparative effectiveness of daytime versus nighttime paravertebral block analgesia on intraoperative hemodynamics in older lung cancer patients: a target trial emulation study
Source: Front Med (Lausanne). 2026 Aug 19;13:1752499. doi: 10.3389/fmed.2026.1752499 (PMC13534014; doi:10.3389/fmed.2026.1752499)
Supplement: Supplementary file 1 [file Supplementary_file_1.DOCX]

**Comparative Effectiveness of Daytime Versus Nighttime Paravertebral Block Analgesia on Intraoperative Hemodynamics in Older Lung Cancer Patients : A Target Trial Emulation Study**

Qiang Wang, Lin Zhuo, Yue Zhang, Bowen Zhang, Kaibing Yang, Shijing Wei, Jingchao Yang, Xinwei Ma, Xiaolin Han, Hui Zheng, Yang Xu

**[eFigure 1.](#_Toc168302539)** [Flowchart of study population.](#_Toc168302539)	**[2](#_Toc168302539)**

**[eFigure 2.](#_Toc168302540)** [Illustration of the proportion of time for hypotension and bradycardia.](#_Toc168302540)	**[3](#_Toc168302540)**

**[eFigure 3.](#_Toc168302541)** [Propensity Score (PS) and IPTW-Weight distribution of two groups.](#_Toc168302541)	**[4](#_Toc168302541)**

**[eFigure 4.](#_Toc168302542)** [Balance of baseline characteristics before and after PSM.](#_Toc168302542)	**[5](#_Toc168302542)**

**[eTable 1.](#_Toc168302543)** [Protocol of a target trial and an emulated trial using real-world data.](#_Toc168302543)	**[6](#_Toc168302543)**

**[eTable 2.](#_Toc168302544)** [Definitions of eligibility criteria.](#_Toc168302544)	**[7](#_Toc168302544)**

**[eTable 3.](#_Toc168302545)** [Definitions of study outcomes.](#_Toc168302545)	**[8](#_Toc168302545)**

**[eTable 4.](#_Toc168302546)** [Baseline characteristics of patients before and after IPTW.](#_Toc168302546)	**[9](#_Toc168302546)**

**[eTable 5.](#_Toc168302547)** [Primary outcomes across subgroups.](#_Toc168302547)	**[10](#_Toc168302547)**

**[eTable 6.](#_Toc168302548)** [Primary outcomes and additional primary outcomes limited to PVB after GA population.](#_Toc168302548)	**[11](#_Toc168302548)**

**[eTable 7.](#_Toc168302549)** [Baseline characteristics of patients after PSM.](#_Toc168302549)	**[12](#_Toc168302549)**

**[eTable 8.](#_Toc168302550)** [Number, rate of and proportion of time for hypotension/bradycardia events in nighttime PVB versus daytime PVB (PSM).](#_Toc168302550)	**[13](#_Toc168302550)**

**[eTable 9.](#_Toc168302551)** [Primary outcomes analysis of different surgical types.](#_Toc168302551)	**[14](#_Toc168302551)**

### eFigure 1. Flowchart of study population.

### eFigure 2. Illustration of the proportion of time for hypotension and bradycardia.

During the surgery period, defined as the time T from the initiation of PVB analgesia to the out of the operating room procedure, the hemodynamics of patient A were measured five times, denoted as Test 1 through Test 5. Hypotension, characterized by systolic blood pressure <90mmHg or diastolic blood pressure <60mmHg, occurred during Tests 1, 3, and 4. Similarly, bradycardia, indicated by a heart rate <60 bpm, was observed during Tests 1, 2, and 3. The time duration of hypotension was calculated as the sum of durations from Tests 1, 3, and 4 (T_1_+T_3_+T_4_). Likewise, the time duration of bradycardia was calculated as the sum of durations from Test 1 to Test 3 (T_1_+T_2_+T_3_). These durations were expressed as proportions of the total surgery duration (T), resulting in (T_1_+T_3_+T_4_) / T and (T_1_+T_2_+T_3_) / T, respectively.

### eFigure 3. Propensity Score (PS) and IPTW-Weight distribution of two groups.

### eFigure 4. Balance of baseline characteristics before and after PSM.

PSM, propensity score matching; SAMD, standardized absolute mean difference; ASA, American Society of Anesthesiologists; SBP, systolic blood pressure; DBP, diastolic blood pressure; Order: the order of paravertebral nerve block analgesia and general anesthesia;

### eTable 1. Protocol of a target trial and an emulated trial using real-world data.

| **Target trial component** | | **Specification** | **Emulation** | |  |
| --- | --- | --- | --- | --- | --- |
| **Causal Estimand** | Eligibility criteria | During a study period from October 19, 2020, to June 28, 2022 for daytime PVB analgesia (8:00-17:00) versus nighttime PVB analgesia (17:00-7:59)  **Inclusion criteria:**   1. elderly patients (≥ 65 years) 2. undergo the lung cancer surgery and receive PVB analgesia. | Same as target trial. Each eligibility criterion is explicitly mapped to corresponding observational data items in **eTable 2**. | |  |
|  |  | **Exclusion criteria:**  with missing information on height, weight, or hemodynamics |  |  |  |
|  | Treatment strategies | **Target trial**   1. Start daytime PVB analgesia and continue use during follow-up 2. Start nighttime PVB analgesia and continue use during follow-up | Same as target trial. | |  |
|  | Assignment (unmasked) | Randomization. Patients are aware to which strategy they are assigned (i.e., no blinding). | Patients are assigned to the treatment strategy their observational data are compatible with at baseline (e.g., patients who filled a daytime PVB analgesia were assigned to the daytime PVB analgesia strategy). | |  |
|  | Outcomes | **Primary outcomes**   1. First and all hypotension 2. First and all bradycardia   **Secondary outcomes**   1. Intravenous fluid use 2. Vasopressors and positive chronotropic agents use | Same as target trial. The study outcome is operationalized for observational data as detailed in **eTable 3.** | |  |
|  | Start/end of follow-up | Follow-up begins after treatment assignment and ends at the occurrence of primary outcome or exiting the operating room. | Same as target trial. | |  |
|  | Causal contrast of interest | Intention-to-treat effect. | Observational analogue of the intention-to-treat. | |  |
| **Identifying Assumptions** | Intention-to-treat effect | Baseline randomization. | Conditional exchangeability assumption for treatment^*^. | |  |
|  | Per-protocol effect | None | None | |  |
| **Estimator** | Intention-to-treat analyses | Patients are analysed according to their original treatment assignment, regardless of treatment discontinuation or switching.  Intention-to-treat effects, measured as HR, IRRs at 0.5 hours intervals, are estimated by Cox proportional hazard regression model and Poisson regression model, respectively. | Baseline randomization is emulated by adjusting for all baseline confounders through inverse probability of treatment weighting.  Intention-to-treat effects, measured as HR, IRRs at 0.5 hours intervals, are estimated by Cox proportional hazard regression model and Poisson regression model, respectively. | |  |
|  | Per-protocol analyses | None | | None | |
|  | Subgroup analyses | - Age (≥70 *vs.* <70 years) - Sex (male *vs.* female) - Baseline hypertension (yes *vs.* no) - Baseline arrhythmia (yes *vs.* no) | Same as the target trial. | |  |
|  | Sensitivity analyses (except per-protocol analyses) | None | To test the robustness of study results influenced by   - Unobserved confounding (violation of conditional exchangeability assumption for treatment^*^):   - E-value   - Different surgical types   - Restricted our study population to patients who received PVB after GA - ATT   - 1:3 PSM | |  |

* The assumption of conditional exchangeability for treatment requires that patients in different treatment strategy groups are exchangeable at time zero conditional on all baseline covariates;

PVB, paravertebral nerve block; IPTW, inverse probability of treatment weighting; GA, general anesthesia; PSM, propensity score matching; ATT, average treatment effect in the treated

### eTable 2. Definitions of eligibility criteria.

| **Eligibility criteria** | **Definition** |
| --- | --- |
| **Inclusion criteria** |  |
| Lung cancer | Preoperative diagnosis includes the word “lung”, including lung masses, lung occupations, lung nodules, lung shadows, and lung cancer in the anesthesia records. |
| PVB | Intraoperative events include “paravertebral” or “PVB”. |
| **Exclusion criteria** |  |
| Height | / |
| Weight | / |
| Hemodynamics | Intraoperative events completely miss blood pressure or heart rate information after PVB analgesia. |

PVB, Paravertebral nerve block.

| **Outcomes** |  | **Type** | **Definition** |
| --- | --- | --- | --- |
| **Primary** |  |  |  |
| Hypotension | First Hypotension | Incident | The first SBP< 90 mmHg or DBP< 60 mmHg during the follow-up |
|  | All Hypotension | Recurrent | Any SBP< 90 mmHg or DBP< 60 mmHg during the follow-up |
| Bradycardia | First Bradycardia | Incident | The first HR< 60 bpm during the follow-up |
|  | All Bradycardia | Recurrent | Any HR< 60 bpm during the follow-up |
| **Additional Primary** |  |  |  |
| Proportion of time for hypotension | | Continuous | The time duration of SBP< 90 mmHg or DBP< 60 mmHg during the follow-up |
| Proportion of time for bradycardia | | Continuous | The time duration of HR< 60 bpm during the follow-up |
| **Secondary** | |  |  |
| Intravenous fluid | Crystalline | Continuous | Patients were given the volume of crystalline fluids intravenously during the follow-up. |
|  | Colloidal | Continuous | Patients were given the volume of colloidal fluids intravenously during the follow-up. |
|  | Total volume | Continuous | Patients were given the volume of crystalline and colloidal fluids intravenously during the follow-up. |
| Medication use | Vasopressors agents | Binary | Patients used ephedrine, methoxamine, or phenylephrine during the follow-up. |
|  | Positive chronotropic agents | Binary | Patients used atropine during the follow-up. |

### eTable 3. Definitions of study outcomes.

SBP, systolic blood pressure; DBP, diastolic blood pressure; HR, heart rate.

### eTable 4. Baseline characteristics of patients before and after IPTW.

|  |  | **Before IPTW** |  |  |  | **After IPTW** |  |
| --- | --- | --- | --- | --- | --- | --- | --- |
| **Patients’ characteristics** | Daytime | Nighttime | SAMD |  | Daytime | Nighttime | SAMD |
|  | (n= 479) | (n= 158) |  |  | (n= 477.7) | (n= 158.62) |  |
| Demographics |  |  |  |  |  |  |  |
| Age, yrs, median [IQR] | 69.00 [67.00, 72.00] | 69.00 [67.00, 71.00] | 0.099 |  | 69.00 [67.00, 72.00] | 69.00 [67.00, 73.00] | 0.034 |
| Female sex, N (%) | 259 (54.1) | 100 (63.3) | 0.188 |  | 268.6 (56.2) | 90.7 (57.2) | 0.019 |
| Height, cm, median [IQR] | 165.00 [159.00, 170.00] | 163.00 [158.00, 170.00] | 0.099 |  | 164.00 [159.00, 170.00] | 163.00 [158.00, 171.00] | 0.016 |
| Weight, kg, median [IQR] | 65.00 [59.00, 73.00] | 65.00 [58.25, 73.00] | 0.062 |  | 65.00 [59.00, 72.96] | 66.00 [59.00, 74.00] | 0.028 |
| Hemodynamics |  |  |  |  |  |  |  |
| SBP, mmHg, median [IQR] | 143.00 [128.00, 154.00] | 145.50 [133.25, 155.00] | 0.104 |  | 143.00 [128.00, 154.00] | 141.21 [130.79, 154.00] | 0.057 |
| DBP, mmHg, median [IQR] | 79.00 [71.00, 86.00] | 78.50 [70.00, 86.00] | 0.004 |  | 78.00 [71.00, 86.00] | 78.00 [69.67, 86.00] | 0.039 |
| Heart rate, bpm, median [IQR] | 72.00 [64.00, 81.00] | 75.00 [67.00, 83.75] | 0.184 |  | 73.00 [64.00, 81.00] | 73.00 [64.04, 83.00] | 0.025 |
| ASA physical status, N (%) |  |  | 0.202 |  |  |  | 0.058 |
| I | 0 (0.0) | 1 (0.6) |  |  | 0.0 (0.0) | 0.2 (0.2) |  |
| II | 442 (92.3) | 151 (95.6) |  |  | 445.4 (93.2) | 148.3 (93.5) |  |
| III | 37 (7.7) | 6 (3.8) |  |  | 32.3 (6.8) | 10.1 (6.4) |  |
| Comorbidities, N (%) |  |  |  |  |  |  |  |
| Hypertension | 200 (41.8) | 49 (31.0) | 0.225 |  | 187.4 (39.2) | 64.3 (40.6) | 0.027 |
| Arrhythmia | 86 (18.0) | 20 (12.7) | 0.147 |  | 79.5 (16.6) | 26.7 (16.8) | 0.005 |
| Hyperthyroidism | 2 (0.4) | 0 (0.0) | 0.092 |  | 1.5 (0.3) | 0.0 (0.0) | 0.079 |
| Hypothyroidism | 1 (0.2) | 2 (1.3) | 0.124 |  | 1.8 (0.4) | 0.7 (0.4) | 0.010 |
| The order of PVB and GA, N (%) |  |  | 0.030 |  |  |  | 0.001 |
| GA after PVB | 10 (2.1) | 4 (2.5) |  |  | 9.8 (2.0) | 3.3 (2.1) |  |
| PVB after GA | 469 (97.9) | 154 (97.5) |  |  | 467.9 (98.0) | 155.4 (97.9) |  |
| Medication history of GA before PVB |  |  |  |  |  |  |  |
| Sedative-hypnotics ^a^, N (%) | 468 (99.8) | 154 (100.0) | 0.065 |  | 467.1 (99.8) | 155.4 (100.0) | 0.059 |
| Analgesics ^b^, N (%) | 468 (99.8) | 154 (100.0) | 0.065 |  | 467.0 (99.8) | 155.4 (100.0) | 0.063 |
| Skeletal muscular relaxants ^c^, N (%) | 469 (100.0) | 154 (100.0) | <0.001 |  | 467.9 (100.0) | 155.4 (100.0) | <0.001 |

Data are reported as frequency (percentage) or median [interquartile range, IQR].

^a^ Propofol, Etomidate and Midazolam

^b^ Sufentanil, Fentanyl and Remifentanil

^c^ Rocuronium and Cisatracurium

Propensity scores were computed by using the following variables: demographics, hemodynamics, ASA physical status, Comorbidities, and the order of PVB and GA.

IPTW, inverse probability of treatment weighting; GA, general anesthesia; PVB, paravertebral nerve block; SAMD, standardized absolute mean difference; ASA, American Society of Anesthesiologists; SBP, systolic blood pressure; DBP, diastolic blood pressure.

### eTable 5. Primary outcomes across subgroups.

| **Subgroup** | | | | | | | | |
| --- | --- | --- | --- | --- | --- | --- | --- | --- |
| **Outcomes** | **First hypotension** | | **All hypotension** | | **First bradycardia** | | **All bradycardia** | |
|  | Adjusted HR (95% CI) | P-value for interaction | Adjusted IRR  (95% CI) | P-value for interaction | Adjusted HR (95% CI) | P-value for interaction | Adjusted IRR (95% CI) | P-value for interaction |
| **Age <70 years** | 0.98 (0.76, 1.27) | 0.99 | 1.11 (1.05, 1.17) | 0.002 | 1.03 (0.78, 1.36) | 0.53 | 0.94 (0.89, 0.99) | <0.001 |
| **Age ≥70 years** | 0.97 (0.74, 1.27) |  | 0.98 (0.92, 1.03) |  | 1.18 (0.87, 1.60) |  | 1.08 (1.03, 1.14) |  |
| **Male** | 0.96 (0.72, 1.29) | 0.93 | 0.93 (0.87, 1.00) | <0.001 | 1.09 (0.77, 1.53) | 0.87 | 0.87 (0.82, 0.93) | <0.001 |
| **Female** | 1.00 (0.79, 1.26) |  | 1.10 (1.05, 1.16) |  | 1.12 (0.88, 1.44) |  | 1.11 (1.06, 1.16) |  |
| **Hypertension no** | 0.87 (0.69, 1.09) | 0.09 | 0.92 (0.87, 0.97) | <0.001 | 1.01 (0.81, 1.27) | 0.33 | 0.94 (0.90, 0.99) | <0.001 |
| **Hypertension yes** | 1.21 (0.89, 1.64) |  | 1.24 (1.17, 1.32) |  | 1.27 (0.85, 1.88) |  | 1.12 (1.06, 1.18) |  |
| **Arrhythmia no** | 0.88 (0.72, 1.08) | 0.01 | 1.02 (0.97, 1.06) | <0.001 | 1.08 (0.87, 1.34) | 0.63 | 1.01 (0.97, 1.05) | 0.69 |
| **Arrhythmia yes** | 1.80 (1.12, 2.89) |  | 1.23 (1.11, 1.36) |  | 1.29 (0.74, 2.24) |  | 1.03 (0.94, 1.13) |  |

CI, confidence interval; HR, hazard ratio; IRR, incidence rate ratio.

### eTable 6. Primary outcomes and additional primary outcomes limited to PVB after GA population.

| Panel A. Hypotension/Bradycardia occurrence (time to first event) | | | | | | | | | | |
| --- | --- | --- | --- | --- | --- | --- | --- | --- | --- | --- |
| **Outcomes** | **Exposure** | | **No. of persons** | **No. of events** | **Median follow-up [IQR], hr** | **Person-hour** | **Incidence rate per p-hr (95% CI)** | | **Crude HR**  **(95% CI)** | **Adjusted HR**  **(95% CI)** |
| First hypotension | Daytime PVB | | 469 | 412 | 1.92 (1.45, 2.47) | 931 | 0.44 (0.10, 2.35) | | Ref. | Ref. |
|  | Nighttime PVB | | 154 | 143 | 1.78 (1.36, 2.35) | 292 | 0.49 (0.11, 2.48) | | 0.99 (0.82,1.19) | 1.00 (0.83, 1.20) |
| First bradycardia | Daytime PVB | | 469 | 374 | 1.92 (1.45, 2.47) | 931 | 0.40 (0.09, 2.23) | | Ref. | Ref. |
|  | Nighttime PVB | | 154 | 127 | 1.78 (1.36, 2.35) | 292 | 0.43 (0.09, 2.33) | | 1.00 (0.82,1.23) | 1.12 (0.91, 1.38) |
| Panel B. Hypotension/Bradycardia recurrence (rate of multiple events per patient) | | | | | | | | | | |
| **Outcomes** | **Exposure** | | **No. of persons** | **No. of events** | **Median follow-up [IQR], hr** | **Person-hour** | **Incidence rate per p-hr (95% CI)** | | **Crude IRR**  **(95% CI)** | **Adjusted IRR**  **(95% CI)** |
| All hypotension | Daytime PVB | | 469 | 3549 | 1.92 (1.45, 2.47) | 931 | 3.81 (1.52, 8.48) | | Ref. | Ref. |
|  | Nighttime PVB | | 154 | 1135 | 1.78 (1.36, 2.35) | 292 | 3.89 (1.56, 8.60) | | 1.03 (0.99, 1.08) | 1.05 (1.01, 1.09) |
| All bradycardia | Daytime PVB | | 469 | 4479 | 1.92 (1.45, 2.47) | 931 | 4.81 (2.09, 9.97) | | Ref. | Ref. |
|  | Nighttime PVB | | 154 | 1368 | 1.78 (1.36, 2.35) | 292 | 4.68 (2.02, 9.78) | | 0.97 (0.94, 1.01) | 1.01 (0.97, 1.05) |
| Panel C. Proportion of time for hypotension/bradycardia | | | | | | | | | | |
| **Outcomes** | **Exposure** | **No. of persons** | | **Mean (SD)** | | **Crude mean difference (95% CI)** | | **Adjusted mean difference (95% CI)** | | |
| Proportion of time for hypotension | Daytime PVB | 469 | | 0.35 (0.28) | | Ref. | | | Ref. | |
|  | Nighttime PVB | 154 | | 0.36 (0.28) | | 0.01 (-0.04, 0.06) | | | 0.01 (-0.04, 0.06) | |
| Proportion of time for bradycardia | Daytime PVB | 469 | | 0.41 (0.35) | | Ref. | | | Ref. | |
|  | Nighttime PVB | 154 | | 0.40 (0.34) | | -0.01 (-0.07, 0.06) | | | 0.01 (-0.05, 0.07) | |

PVB, paravertebral nerve block; CI, confidence interval; p-hr, person-hour; HR, hazard ratio; IRR, incidence rate ratio; SD, standard deviation.

Number of events, person-hour, incidence rates and mean were calculated in the unweighted population.

***eTable 7. Baseline characteristics of patients after PSM.***

|  |  | **After PSM** |  |
| --- | --- | --- | --- |
| **Patients’ characteristics** | Daytime | Nighttime | SAMD |
|  | (n= 357) | (n= 148) |  |
| Demographics |  |  |  |
| Age, yrs, median [IQR] | 69.00 [67.00, 72.00] | 69.00 [67.00, 71.25] | 0.021 |
| Female sex, N (%) | 211 (59.1) | 91 (61.5) | 0.049 |
| Height, cm, median [IQR] | 163.00 [158.00, 170.00] | 163.00 [158.00, 170.25] | 0.006 |
| Weight, kg, median [IQR] | 65.00 [58.00, 72.00] | 65.00 [58.00, 73.00] | 0.010 |
| Hemodynamics |  |  |  |
| SBP, mmHg, median [IQR] | 143.00 [129.00, 155.00] | 145.50 [133.00, 155.25] | 0.054 |
| DBP, mmHg, median [IQR] | 78.00 [72.00, 85.00] | 80.00 [70.00, 86.25] | 0.024 |
| Heart rate, bpm, median [IQR] | 73.00 [65.00, 81.00] | 74.00 [67.00, 83.00] | 0.074 |
| ASA physical status, N (%) |  |  | 0.034 |
| I | 0 (0.0) | 0 (0.0) |  |
| II | 340 (95.2) | 142 (95.9) |  |
| III | 17 (4.8) | 6 (4.1) |  |
| Comorbidities, N (%) |  |  |  |
| Hypertension | 131 (36.7) | 48 (32.4) | 0.090 |
| Arrhythmia | 54 (15.1) | 20 (13.5) | 0.046 |
| Hyperthyroidism | 0 (0.0) | 0 (0.0) | <0.001 |
| Hypothyroidism | 0 (0.0) | 0 (0.0) | <0.001 |
| The order of PVB and GA, N (%) |  |  | 0.076 |
| GA after PVB | 7 (2.0) | 3 (2.0) | 0.005 |
| PVB after GA | 350 (98.0) | 145 (98.0) |  |
| Medication history of GA before PVB |  |  |  |
| Sedative-hypnotics ^a^, N (%) | 350 (100.0) | 145 (100.0) | <0.001 |
| Analgesics ^b^, N (%) | 350 (100.0) | 145 (100.0) | <0.001 |
| Skeletal muscular relaxants ^c^, N (%) | 350 (100.0) | 145 (100.0) | <0.001 |

Data are reported as frequency (percentage) or median [interquartile range, IQR].

^a^ Propofol, Etomidate and Midazolam

^b^ Sufentanil, Fentanyl and Remifentanil

^c^ Rocuronium and Cisatracurium

Propensity scores were computed by using the following variables: demographics, hemodynamics, ASA physical status, Comorbidities, and the order of PVB and GA.

PSM, propensity score matching; GA, general anesthesia; PVB, paravertebral nerve block; SAMD, standardized absolute mean difference; ASA, American Society of Anesthesiologists; SBP, systolic blood pressure; DBP, diastolic blood pressure.

### eTable 8. Number, rate of and proportion of time for hypotension/bradycardia events in daytime PVB versus nighttime PVB (PSM).

| Panel A. Hypotension/Bradycardia occurrence (time to first event) | | | | | | | | | | |
| --- | --- | --- | --- | --- | --- | --- | --- | --- | --- | --- |
| **Outcomes** | **Exposure** | | **No. of persons** | **No. of events** | **Median follow-up [IQR], hr** | **Person-hour** | **Incidence rate per p-hr (95% CI)** | **Crude HR**  **(95% CI)** | | **Adjusted HR**  **(95% CI)** |
| First hypotension | Daytime PVB | | 479 | 422 | 1.93 (1.47, 2.48) | 958 | 0.44 (0.10, 2.34) | Ref. | | Ref. |
|  | Nighttime PVB | | 158 | 147 | 1.78 (1.37, 2.35) | 300 | 0.49 (0.11, 2.48) | 0.99 (0.82,1.20) | | 0.91 (0.71, 1.17) |
| First bradycardia | Daytime PVB | | 479 | 382 | 1.93 (1.47, 2.48) | 958 | 0.40 (0.09, 2.22) | Ref. | | Ref. |
|  | Nighttime PVB | | 158 | 130 | 1.78 (1.37, 2.35) | 300 | 0.43 (0.09, 2.32) | 1.00 (0.82,1.21) | | 1.11 (0.86, 1.42) |
| Panel B. Hypotension/Bradycardia recurrence (rate of multiple events per patient) | | | | | | | | | | |
| **Outcomes** | **Exposure** | | **No. of persons** | **No. of events** | **Median follow-up [IQR], hr** | **Person-hour** | **Incidence rate per p-hr (95% CI)** | **Crude IRR**  **(95% CI)** | | **Adjusted IRR**  **(95% CI)** |
| All hypotension | Daytime PVB | | 479 | 3678 | 1.93 (1.47, 2.48) | 958 | 3.84 (1.53, 8.52) | Ref. | | Ref. |
|  | Nighttime PVB | | 158 | 1170 | 1.78 (1.37, 2.35) | 300 | 3.90 (1.57, 8.61) | 1.04 (1.01, 1.08) | | 1.05 (1.01, 1.10) |
| All bradycardia | Daytime PVB | | 479 | 4558 | 1.93 (1.47, 2.48) | 958 | 4.76 (2.06, 9.89) | Ref. | | Ref. |
|  | Nighttime PVB | | 158 | 1410 | 1.78 (1.37, 2.35) | 300 | 4.70 (2.02, 9.81) | 0.98 (0.95, 1.02) | | 1.03 (0.99, 1.07) |
| Panel C. Proportion of time for hypotension/bradycardia | | | | | | | | | | |
| **Outcomes** | **Exposure** | **No. of persons** | | **Mean (SD)** | | **Crude mean difference (95% CI)** | | | **Adjusted mean difference (95% CI)** | |
| Proportion of time for hypotension | Daytime PVB | 479 | | 0.35 (0.28) | | Ref. | | Ref. | | |
|  | Nighttime PVB | 158 | | 0.36 (0.28) | | 0.01 (-0.04, 0.06) | | 0.02 (-0.04, 0.07) | | |
| Proportion of time for bradycardia | Daytime PVB | 479 | | 0.41 (0.35) | | Ref. | | Ref. | | |
|  | Nighttime PVB | 158 | | 0.40 (0.34) | | -0.003 (-0.064, 0.059) | | 0.02 (-0.04, 0.08) | | |

PVB, paravertebral nerve block; CI, confidence interval; p-hr, person-hour; HR, hazard ratio; IRR, incidence rate ratio; SD, standard deviation.

Number of events, person-hour, incidence rates and mean were calculated in the unmatched population.

### eTable 9. Primary outcomes analysis of different surgical types.

|  | **First hypotension** | | **All hypotension** | | **First bradycardia** | | **All bradycardia** | |
| --- | --- | --- | --- | --- | --- | --- | --- | --- |
| **Subgroups** | Adjusted HR (95% CI) | P-value for interaction | Adjusted IRR  (95% CI) | P-value for interaction | Adjusted HR (95% CI) | P-value for interaction | Adjusted IRR (95% CI) | P-value for interaction |
| **Thoracotomy** | 0.82 (0.37, 1.83) | 0.70 | 0.74 (0.61, 0.90) | <0.001 | 0.99 (0.45, 2.21) | 0.83 | 1.07 (0.91, 1.26) | 0.48 |
| **Video thoracoscopy** | 1.00 (0.83, 1.21) |  | 1.06 (1.02, 1.11) |  | 1.11 (0.90, 1.37) |  | 1.01 (0.97, 1.05) |  |

CI, confidence interval; HR, hazard ratio; IRR, incidence rate ratio.
